# Physical activity and sedentary behavior patterns using accelerometry from a national sample of United States adults

**DOI:** 10.1186/s12966-015-0183-7

**Published:** 2015-02-15

**Authors:** Kelly R Evenson, Fang Wen, Jesse S Metzger, Amy H Herring

**Affiliations:** Department of Epidemiology, Gillings School of Global Public Health, University of North Carolina Chapel Hill, 137 East Franklin Street, Suite 306, Chapel Hill, NC USA; Center for Behavioral Health Research and Services, University of Alaska – Anchorage, Anchorage, AK USA; Department of Biostatistics, Gillings School of Global Public Health, Carolina Population Center, University of North Carolina – Chapel Hill, Chapel Hill, NC USA

**Keywords:** Accelerometry, Latent class analysis, Moderate to vigorous physical activity, Surveillance, Weekend warrior

## Abstract

**Background:**

This study described the patterns of accelerometer-determined physical activity and sedentary behavior among adults using a nationally representative sample from the United States.

**Methods:**

Using 2003-2006 National Health and Nutrition Examination Survey (NHANES) data, 7931 adults at least 18 years old wore an ActiGraph accelerometer for one week, providing at least 3 days of wear for >=8 hours/day. Cutpoints defined moderate to vigorous physical activity (MVPA; >= 2020 and >=760 counts/minute), vigorous physical activity (> = 5999 counts/minute), and sedentary behavior (<100 counts/minute). Latent class analysis (LCA) was used to estimate patterns of physical activity and sedentary behavior. All estimates were weighted to reflect the United States population.

**Results:**

For weighted percent of MVPA out of total wearing time, 5 classes were identified from least to most active: 65.3% of population (weighted mean 9.3 minutes/day), 24.9% (32.1 minutes/day), 3.2% that was low on the weekdays but much higher on the weekends (52.0 minutes/day), 5.9% (59.9 minutes/day), and 0.7% in the highest class (113.6 minutes/day). Using the lower MVPA threshold, 6 classes emerged with each class ranging in population from 1.2% to 43.6%. A vigorous activity class could not be derived due to low prevalence. For weighted percent of sedentary behavior out of total wearing time, 5 classes were identified from most to least sedentary: 6.3% of population (weighted mean 660.2 minutes/day), 25.1% (546.8 minutes/day), 37.7% (453.9 minutes/day), 24.0% (354.8 minutes/day), and 7.0% (256.3 minutes/day). Four of the classes showed generally similar results across every day of the week, with the absolute percents differing across classes. In contrast, the least sedentary class showing a marked rise in percent of time spent in sedentary behavior on the weekend (weighted mean 336.7-346.5 minutes/day) compared to weekdays (weighted mean 255.2-292.4 minutes/day).

**Conclusion:**

The LCA models provided a data reduction process to identify patterns using minute-by-minute accelerometry data in order to explore meaningful contrasts. The models supported 5 or 6 distinct patterns for MVPA and sedentary behavior. These physical activity and sedentary behavior patterns can be used as intervention targets and as independent or dependent variables in future studies of correlates, determinants, or outcomes.

**Electronic supplementary material:**

The online version of this article (doi:10.1186/s12966-015-0183-7) contains supplementary material, which is available to authorized users.

## Introduction

In 2008, the United States government released its first physical activity recommendations [1] about the types and amounts of physical activity recommended to offer substantial health benefits to all Americans. The guidelines were based, in part, on epidemiologic studies of health outcomes, including all-cause and cardiovascular disease mortality. Those studies relied almost exclusively on self-reported physical activity. Self-reported measures, such as questionnaires, have a limited ability to detect physical activity that is routine and interspersed throughout the day, such as unstructured activities. These tend to be activities that are light or sedentary. As a complement to self-report, accelerometers can provide detailed measures of time spent in both physical activity and sedentary behavior.

Prior epidemiologic work using accelerometry typically categorizes physical activity into number of minutes or bout minutes (defined as extended periods of time in a certain level of intensity). While this grouping is useful, it ignores potential differences in the patterns of accumulated physical activity over time. For example, one weekly pattern of physical activity to emerge from self-reported questionnaires is the “weekend warrior” [2,3]. This pattern is characterized by accumulation of a high total volume of physical activity during the weekend and much less total volume on the weekdays. Lee et al. [2] quantified this as at least 1000 kilocalories from sports or recreational activities over 1-2 days/week, while Kruger et al. [3] quantified this as at least 150 minutes of moderate to vigorous physical activity (MVPA)/week performed on 1-2 days/week. Accelerometry can provide information even down to the second on physical activity and sedentary behavior, allowing for more precise exploration into the patterning of these behaviors.

Latent class analysis (LCA) is a method that can be applied to accelerometry, whereby participants are assumed to belong to one of several mutually exclusive classes, but for which class membership is not known a priori. Through a statistical model, the latent class analysis assigns participants to a category (class) based on the associations among observed variables. This technique provides a method to identify patterns of physical activity classes, such as the weekend warrior class. Thus far, only a few accelerometry studies of adults have applied LCA techniques [4,5] and we found no LCA studies of adults that explored sedentary behavior patterns. Thus, this study employed LCA techniques to describe patterns of accelerometer-assessed physical activity and sedentary behavior among a national sample of US adults. The resulting patterns were described by age, gender, and race/ethnicity to understand how the patterns varied by sociodemographic characteristics.

## Methods

### Data sources

Through in-person interviews and physical examinations, the National Health and Nutrition Examination Survey (NHANES) provides a cross-sectional assessment of nutrition and health of the US population. The data used in this study were obtained during 2003 to 2006, the most recently available data with accelerometer assessed physical activity. Participants provided informed consent before completing any questionnaires or measurements. The overarching project was reviewed by the University of North Carolina Institutional Review Board.

### Physical activity measurement by accelerometry

Those who participated in the physical activity monitor examination were asked to wear the ActiGraph accelerometer (model #AM7164) on their hip for seven consecutive days during waking hours and outside of any water-based activities. Beginning at midnight on the day following the clinic visit, the accelerometer recorded 1-minute epochs of analog acceleration and converted it to a digital signal [6]. Non-wear was defined by an interval of at least 90 consecutive minutes of zero counts/minute, with allowance of 1 or 2 minutes of nonzero counts if no counts were detected during both the 30 minutes upstream and downstream from that interval; any nonzero counts except the allowed short intervals were considered as wear time [7]. Counts in the non-wear period were set to missing.

The ActiGraph accelerometer assessed acceleration using counts as the output metric. To interpret counts, researchers use cutpoints or thresholds to characterize activities by intensity, which includes sedentary, light, moderate, or vigorous activity. We used cutpoints originally applied to NHANES [8]. Vigorous intensity was defined as > =5999 counts/minute and moderate intensity as 2020-5998 counts/minute. This higher cutpoint approximates moderate activity based primarily on treadmill walking or running. A lower moderate intensity threshold was calculated based on studies that incorporated more lifestyle activities, defined as > =760 counts/minute [9]. We refer to these two MPVA cutpoints based on the first author’s last names (Troiano and Matthews, respectively). Another type of MVPA was categorized based on time spent in MVPA bouts, separately for the Troiano and Matthews cutpoints, with a bout defined as at least 10 minutes of consecutive MVPA with allowance for interruptions of up to 20% below the threshold and with <5 consecutive minutes below the threshold. A MVPA bout also had to start and end with a count over the threshold.

Sedentary behavior was defined as <100 counts/minute [10]. Sedentary bouts were defined as > =30 minutes with at least 80% of the minutes falling below the sedentary threshold and with <5 consecutive minutes above the threshold [11]. A sedentary bout had to start and end with a count below the threshold.

### Other measures

Self-reported sociodemographic measures used in this study included age, gender, and race/ethnicity (Non-Hispanic White, Non-Hispanic Black, Hispanic, other). In the NHANES data, participants age 85 or older were top coded to age 85 in order to protect their confidentiality. We explored age in categories, so this categorization did not affect our results.

### Statistical methods

The sample was limited to those age 18 years and older (n = 11,183), who participated in the accelerometer portion of NHANES during 2003-2006 (n = 9601). We further excluded 619 participants whose accelerometer was not in calibration or was faulty upon return (i.e., recording no counts) and 1051 who did not provide at least 3 days of accelerometer wear for 8 or more hours per day over a seven-day period. This left a final sample size of 7931 in which 5.8% wore it three days (n = 459), 8.1% wore it four days (n = 645), 12.6% wore it five days (n = 998), 21.9% wore it six days (n = 1740), and 51.6% wore it 7 days (n = 4089). In addition, we explored the latent class analyses only among those who contributed two adherent weekend days (and therefore at least one adherent weekday; n = 5430).

To account for the differential probability of selection, all percents and means were weighted to the 2000 census using the 4-year sample weights provided by NHANES. The data were nested (i.e., screener, household interview, examination), such that non-response and post-stratification adjustments were applied.

Using LCA, we used up to 7 adherent days from the participant’s accelerometer file to determine classes, or natural groupings, of participants who tended to accumulate their physical activity or sedentary behavior in a similar pattern. The derived classes were among participants who shared similar means, separately calculated for the following indicators (all weighted):

- counts/minute per day (an indicator of total volume),
- percent of MVPA out of total wearing time per day (using both the Troiano and Matthews cutpoints),
- percent of MVPA bouts out of total wearing time per day (using both the Troiano and Matthews cutpoints),
- percent of sedentary behavior out of total wearing time per day, and
- percent of sedentary bouts out of total wearing time per day.

While we explored using absolute minutes (both with and without control for wearing time), the final classes for MVPA and sedentary behavior were based on relative percents in order to best account for accelerometer wearing time. We also conducted analyses among a subset of participants with both weekend days, in order to make sure no unique pattern was missed due to non-wear. In the end, this only impacted sedentary bouts, for which more classes emerged as a result. We were unable to derive percent of time in vigorous activity out of total wearing time due to few participants engaging in vigorous activity.

Several criteria were used to select the final number of classes for each physical activity or sedentary behavior variable. These criteria included:

1. the bootstrap likelihood ratio test, which compared the fit of k classes to (k-1) classes,
2. sample size of the classes, requiring each class to have no fewer than 50 participants, and
3. substantive knowledge, including a practical interpretation of what each class represented, along with visual inspection, to ensure that the classes were sufficiently separated from each other (entropy).

The LCA was performed using MPlus (version 7.11) [12], which allowed for the complex survey design in conjunction with the modeling. Mixture modelling was applied to describe the relationship between up to 7 adherent days of accelerometry and the categorical latent variable using a set of linear regression equations. Due to the large number of participants with zero for percent of MVPA bouts out of total wearing time (Troiano or Matthews), the LCA with zero-inflated negative binomial models were used.

For each variable, a 4-class model was estimated first, based on two prior NHANES analyses [4,5]. We also examined 3-class models and continued models up to 12 classes if necessary, but stopped at this point since the sample sizes of the most active and most sedentary classes usually became too small. Each participant was assigned to one class based on the highest posterior class membership probability (modal allocation), separately for each variable. Using SAS® release 9.3 (Cary, North Carolina), classes were explored using the weighted means of each variable by day of the week and by accelerometer wear time. Weighted means of class assignments were also calculated for all variables overall and by age, gender, and race/ethnicity. Spearman correlation coefficients were calculated to compare the two MVPA definitions.

## Results

**Figure 1:**
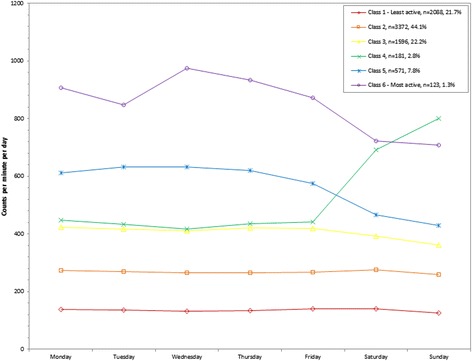
**Latent class analysis plotted for weighted mean counts/minute; NHANES 2003-2006.**

**Figure 2:**
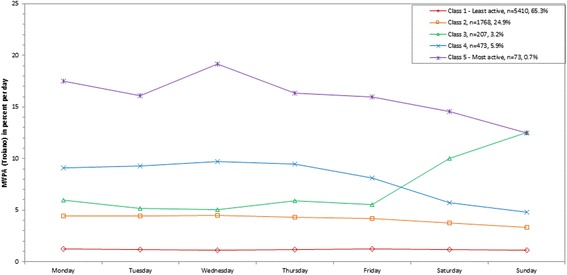
**Latent class analysis plotted for weighted percent of moderate to vigorous physical activity (MVPA; Troiano) out of total wearing time per day; NHANES 2003-2006.**

**Figure 3:**
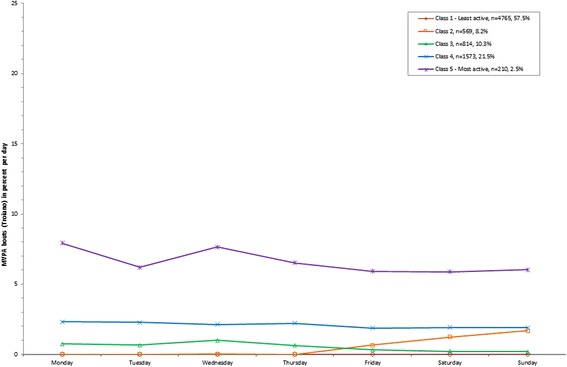
**Latent class analysis plotted for weighted percent of moderate to vigorous physical activity (MVPA) bouts (Troiano) out of total wearing time per day; NHANES 2003-2006.**

**Figure 4:**
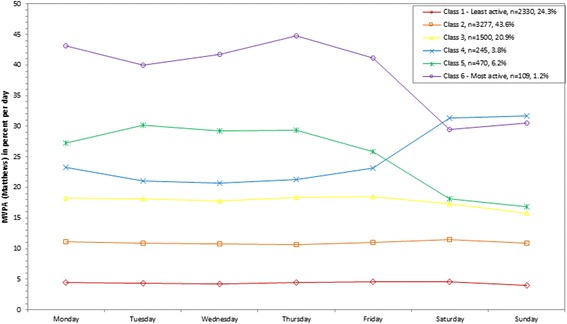
**Latent class analysis plotted for weighted percent of moderate to vigorous physical activity (MVPA; Matthews) out of total wearing time per day; NHANES 2003-2006.**

**Figure 5:**
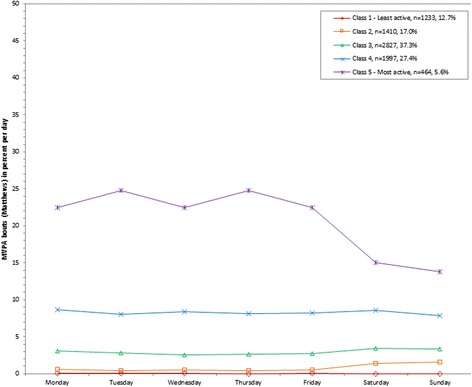
**Latent class analysis plotted for weighted percent of moderate to vigorous physical activity (MVPA) bouts (Matthews) out of total wearing time per day; NHANES 2003-2006.**

**Figure 6:**
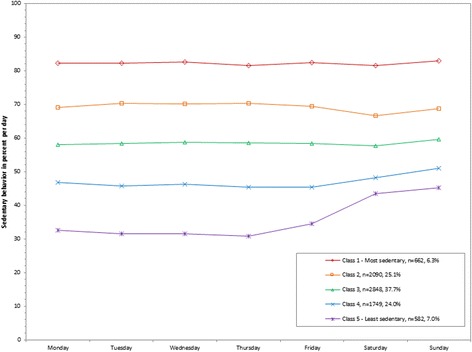
**Latent class analysis plotted for weighted percent of sedentary behavior out of total wearing time per day; NHANES 2003-2006.**

**Figure 7:**
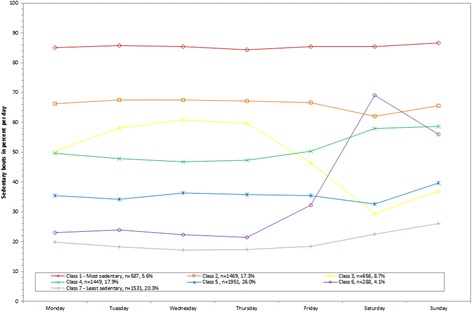
**Latent class analysis plotted for weighted percent of sedentary bouts out of total wearing time per day; NHANES 2003-2006.**

Participants were classified into 5 classes for percent of MVPA out of total wearing time per day (Troiano), percent of MVPA bouts out of total wearing time per day (Troiano and Matthews), and percent of sedentary behavior out of total wearing time per day. Using linear regression the bootstrap likelihood ratio test was <0.001 for each variable. Participants were classified into 6 classes for counts/minute and percent of MVPA out of total wearing time per day (Matthews), and 7 classes for percent of sedentary bouts out of total wearing time per day. The bootstrap likelihood ratio test was <0.001 for each variable. For all variables, the graphs of the classes by day are shown in Figures 1, 2, 3, 4, 5, 6 and 7 and the weighted mean percents by day are detailed in Additional file 1.

For each variable, we explored accelerometer wearing time overall and by day within each derived class. For all seven variables, weighted mean weekly accelerometer wear time ranged from 13.3 to 14.6 hours/day by derived class. Generally longer mean wear was documented for classes with less MVPA and more sedentary behavior (Additional file 2).

### Total volume

An indicator of total volume of physical activity ranged from a weighted mean of 135.4 (class 1) to 852.4 (class 6) counts/minute across the 6 classes (Table 1). The two most active classes represented 9.1% of the population and indicated stable higher weighted means on the weekdays (class 5: 575.6 (Friday) to 631.7 (Tuesday) counts/minute; class 6: 846.8 (Tuesday) to 974.9 (Wednesday) counts/minute) and lower on the weekends (class 5: 429.4 (Sunday) to 465.3 (Saturday) counts/minute; class 6: 707.9 (Sunday) to 723.6 (Saturday) counts/minute) (Figure 1). A unique class emerged with lower weighted means on the weekdays (class 4: 417.5 (Wednesday) to 448.2 (Monday) counts/minute) and higher on the weekends (692.2 (Saturday) to 801.5 (Sunday) counts/minute). The remaining three classes (class 1-3) were relatively stable across the week and had the lowest weighted mean counts/minute.

**Table 1:** **Latent classes derived from accelerometry among adults (n = 7931); NHANES 2003-2006**

				**Weighted mean of average counts/minute, MVPA (minutes/day), or sedentary behavior (minutes/day)**							
	**n**	**Weighted percent of population in class**	**Average percent out of total wearing time**	**Overall**	**Monday**	**Tuesday**	**Wednesday**	**Thursday**	**Friday**	**Saturday**	**Sunday**
Average counts/minute											
Class 1 - Least active	2088	21.7		135.4	138.1	135.3	131.6	134.6	139.8	139.6	125.6
Class 2	3372	44.1		267.2	272.5	269.0	265.9	265.2	266.2	274.6	257.9
Class 3	1596	22.2		406.0	423.9	416.7	410.7	421.9	418.4	391.7	361.6
Class 4	181	2.8		510.2	448.2	432.9	417.5	436.2	442.3	692.2	801.5
Class 5	571	7.8		568.2	611.5	631.7	631.4	620.6	575.6	465.3	429.4
Class 6 - Most active	123	1.3		852.4	906.5	846.8	974.9	934.6	873.2	723.6	707.9
Average percent of MVPA (Troiano) out of total wearing time per day											
Class 1 - Least active	5410	65.3	1.2	9.3	10.8	10.4	9.9	10.3	10.5	10.0	8.9
Class 2	1768	24.9	4.2	32.1	38.5	39.0	38.8	37.6	36.6	31.0	26.8
Class 3	207	3.2	7.1	52.0	49.8	44.8	43.3	51.4	47.5	78.4	95.2
Class 4	473	5.9	8.2	59.9	77.3	78.2	82.0	78.3	68.5	47.6	39.0
Class 5 - Most active	73	0.7	16.1	113.6	150.4	138.2	159.6	133.0	127.6	112.1	92.1
Percent of MVPA bouts (Troiano) out of total wearing time per day											
Class 1 - Least active	4765	57.5	0.0	0.0	0.1	0.1	0.0	0.2	0.0	0.0	0.0
Class 2	569	8.2	0.5	4.3	0.0	0.0	0.2	0.0	5.8	10.2	13.3
Class 3	814	10.3	0.5	4.9	6.6	6.0	9.0	5.6	3.0	1.9	1.6
Class 4	1573	21.5	2.1	17.8	19.8	19.9	18.4	19.3	16.3	15.9	15.4
Class 5 - Most active	210	2.5	6.7	53.3	66.5	51.1	63.4	53.3	47.8	46.0	45.0
Percent of MVPA (Matthews) out of total wearing time per day											
Class 1 - Least active	2330	24.3	4.4	34.6	37.8	37.6	36.5	37.7	39.6	38.7	33.2
Class 2	3277	43.6	11.0	84.3	97.0	95.8	94.4	92.6	95.7	94.4	86.6
Class 3	1500	20.9	17.8	133.9	156.0	153.5	153.0	155.9	157.5	141.0	126.7
Class 4	245	3.8	24.6	183.6	198.1	185.4	178.6	184.2	195.9	251.6	246.4
Class 5	470	6.2	25.8	185.1	229.6	255.1	245.0	244.7	218.0	152.7	129.0
Class 6 - Most active	109	1.2	39.5	266.3	358.6	334.6	341.8	352.2	328.7	226.9	237.0
Percent of MVPA bouts (Matthews) out of total wearing time per day											
Class 1 - Least active	1233	12.7	0.0	0.2	0.3	0.3	0.3	0.2	0.3	0.0	0.0
Class 2	1410	17.0	0.8	6.4	5.1	3.9	4.8	3.7	4.8	11.4	12.5
Class 3	2827	37.3	2.9	24.8	26.6	24.4	22.2	23.4	24.1	28.8	26.9
Class 4	1997	27.4	8.3	69.6	74.0	69.0	72.2	69.7	70.7	70.1	62.5
Class 5 - Most active	464	5.6	21.3	175.4	189.2	212.4	190.6	203.7	185.4	124.1	109.8
Percent of sedentary behavior out of total wearing time per day											
Class 1 - Most sedentary	662	6.3	82.3	660.2	732.5	736.5	725.2	725.6	728.6	726.3	735.4
Class 2	2090	25.1	69.3	546.8	606.6	624.3	616.3	617.5	613.3	562.6	568.8
Class 3	2848	37.7	58.4	453.9	502.1	507.1	512.6	505.1	509.6	479.5	483.5
Class 4	1749	24.0	46.7	354.8	399.3	387.9	394.1	384.7	388.2	395.0	404.3
Class 5 - Least sedentary	582	7.0	35.0	256.3	271.8	263.0	261.1	255.2	292.4	346.5	336.7
Percent of sedentary bouts out of total wearing time per day											
Class 1 - Most sedentary	587	5.6	85.6	746.7	760.6	761.5	749.0	749.1	751.0	757.7	767.6
Class 2	1469	17.3	66.2	568.9	578.5	597.4	591.5	587.4	585.0	527.6	548.5
Class 3	656	8.7	49.2	425.7	448.0	518.5	534.8	523.0	420.9	246.1	302.1
Class 4	1449	17.9	51.0	441.0	436.8	426.1	416.1	418.2	446.1	490.0	486.1
Class 5	1951	26.0	35.6	300.8	305.2	293.6	315.9	306.6	308.2	274.4	323.0
Class 6	288	4.1	35.1	292.1	204.8	208.2	195.4	186.6	288.2	551.3	441.9
Class 7 - Least sedentary	1531	20.3	19.5	159.0	166.0	153.5	144.8	146.3	156.5	181.7	199.8

MVPA = moderate to vigorous physical activity.

Females had higher representation in the least active two classes for weighted mean counts/minute (61.0% class 1, 58.9% class 2; Table 2). Males were much more likely to be represented in the most active three classes (68.5% to 81.4%). In the least active class, adults > =65 years (48.4%) and Non-Hispanic Whites (76.9%) had much higher representation compared to other categories. Adults 18-34 were over represented the most active class (49.3%).

**Table 2:** **Sociodemographic characteristics by accelerometry derived classes among adults (n = 7931); NHANES 2003-2006**

		**Weighted percent: gender**		**Weighted percent: age in years**				**Weighted percent: race/ethnicity**			
	**n**	**Female (n = 4118)**	**Male (n = 3813)**	**18-34 (n = 2530)**	**35-49 (n = 1790)**	**50-64 (n = 1627)**	**> = 65 (n = 1984)**	**Non-Hispanic White (n = 3953)**	**Non-Hispanic Black (n = 1727)**	**Hispanic (N = 1927)**	**Other (N = 324)**
Average counts/minute											
Class 1 - Least active	2088	61.0	39.0	10.9	15.5	25.2	48.4	76.9	11.0	6.1	6.0
Class 2	3372	58.9	41.1	27.7	31.6	28.3	12.4	72.2	11.8	10.2	5.7
Class 3	1596	46.5	53.5	35.1	37.9	21.7	5.2	71.4	10.4	14.0	4.2
Class 4	181	31.5	68.5	40.2	42.6	15.6	1.6	79.3	6.0	6.9	7.8
Class 5	571	25.0	75.0	42.0	39.9	15.1	3.0	67.2	7.2	22.4	3.3
Class 6 - Most active	123	18.6	81.4	49.3	40.7	9.1	0.9	48.2	13.2	34.5	4.1
Percent of MVPA (Troiano) out of total wearing time per day											
Class 1 - Least active	5410	61.6	38.4	21.4	26.7	27.9	23.9	73.3	11.4	9.5	5.7
Class 2	1768	40.1	59.9	37.2	37.4	19.4	6.1	72.6	9.9	13.4	4.2
Class 3	207	26.6	73.4	37.3	46.1	14.3	2.3	78.4	5.3	9.1	7.3
Class 4	473	25.4	74.6	44.8	35.8	15.8	3.5	63.8	10.9	20.5	4.8
Class 5 - Most active	73	16.6	83.4	55.1	32.7	12.2	0.0	44.0	13.5	40.1	2.4
Percent of MVPA bouts (Troiano) out of total wearing time per day											
Class 1 - Least active	4765	58.0	42.0	22.7	27.7	26.1	23.6	73.2	11.6	9.7	5.6
Class 2	569	43.7	56.3	31.6	36.7	23.1	8.5	74.8	7.8	13.3	4.1
Class 3	814	45.1	54.9	34.2	32.4	23.6	9.8	71.0	10.7	13.8	4.5
Class 4	1573	47.8	52.2	34.2	35.1	21.5	9.3	71.5	9.9	13.1	5.5
Class 5 - Most active	210	32.6	67.4	39.1	30.9	23.4	6.7	64.9	12.7	17.8	4.7
Percent of MVPA (Matthews) out of total wearing time per day											
Class 1 - Least active	2330	62.7	37.3	12.4	15.7	25.0	46.9	76.0	11.2	6.4	6.4
Class 2	3277	57.6	42.4	29.6	32.2	27.8	10.4	72.7	11.6	10.3	5.4
Class 3	1500	44.4	55.6	34.3	38.2	21.6	5.9	72.9	10.3	13.1	3.7
Class 4	245	32.6	67.4	34.8	45.5	17.1	2.7	71.2	7.2	11.6	10.0
Class 5	470	26.1	73.9	39.5	41.1	16.9	2.5	63.2	8.6	25.5	2.8
Class 6 - Most active	109	17.4	82.6	48.6	37.3	13.3	0.8	44.1	7.1	46.2	2.7
Percent of MVPA bouts (Matthews) out of total wearing time per day											
Class 1 - Least active	1233	66.2	33.8	12.2	15.4	22.3	50.1	77.7	11.0	6.8	4.5
Class 2	1410	66.1	33.9	23.6	26.2	29.1	21.1	71.2	12.7	8.2	7.8
Class 3	2827	54.5	45.5	29.5	32.1	25.4	13.0	73.0	10.9	10.6	5.6
Class 4	1997	41.5	58.5	31.5	36.5	23.1	8.9	73.8	9.8	12.6	3.8
Class 5 - Most active	464	23.5	76.5	41.0	38.9	16.7	3.4	55.6	9.1	30.6	4.8
Percent of sedentary behavior out of total wearing time per day											
Class 1 - Most sedentary	662	49.7	50.3	11.4	11.1	20.2	57.4	79.8	10.7	4.0	5.5
Class 2	2090	51.2	48.8	23.8	25.0	25.4	25.7	75.5	11.4	7.2	6.0
Class 3	2848	57.5	42.5	27.1	31.3	27.2	14.4	73.8	11.4	8.8	5.9
Class 4	1749	53.0	47.0	32.9	37.3	22.5	7.3	70.0	9.4	16.5	4.1
Class 5 - Least sedentary	582	33.2	66.8	38.5	41.1	18.0	2.3	57.1	10.6	28.7	3.6
Percent of sedentary bouts out of total wearing time per day											
Class 1 - Most sedentary	587	48.5	51.5	9.6	8.4	18.3	63.8	81.0	9.9	3.0	6.1
Class 2	1469	47.7	52.3	20.9	24.2	25.3	29.5	76.8	10.9	6.1	6.2
Class 3	656	57.7	42.3	30.8	33.6	25.4	10.2	73.9	11.3	9.1	5.6
Class 4	1449	54.4	45.6	25.8	25.9	26.9	21.4	73.4	12.3	9.2	5.1
Class 5	1951	54.6	45.4	29.0	33.5	26.8	10.7	73.0	10.6	11.1	5.3
Class 6	288	48.0	52.0	28.4	38.3	27.1	6.2	69.4	10.4	13.8	6.4
Class 7 - Least sedentary	1531	52.9	47.1	35.8	39.6	19.8	4.8	65.2	9.9	20.8	4.1

MVPA = moderate to vigorous physical activity.

Note: row percents are presented by category.

### MVPA

MVPA was explored using two definitions: a higher cutpoint termed “Troiano” and a lower cutpoint termed “Matthews”. The Spearman correlation between the two was 0.62 for MVPA minutes/day and 0.57 for MVPA bout minutes/day.

The weighted mean percent of MVPA (Troiano) out of total wearing time per day ranged from 1.2% (class 1) for the least active class to 16.1% (class 5) for the most active class (9.3 to 113.6 minutes/day) (Table 1). A weekend warrior class emerged for 3.2% of the population (class 3; Figure 2), with a weighted mean MVPA of 43.3 (Wednesday) to 51.4 (Thursday) minutes/day on weekdays, 78.4 on Saturday, and 95.2 on Sunday. The most active class emerged for 0.7% of the population (class 5), with a higher percent of MVPA out of total wearing time on the weekdays (127.6 (Friday) to 159.6 (Wednesday) minutes/day) and a lower percent on the weekends (92.1 (Sunday) to 112.1 (Saturday) minutes/day), but still high relative to all other classes. A parallel class but with lower absolute percentages also emerged for 5.9% of the sample (class 4), with a higher percent of MVPA out of total wearing time on the weekdays (68.5 (Friday) to 82.0 (Wednesday) minutes/day) and a lower percent on the weekends (39.0 (Sunday) to 47.6 (Saturday) minutes/day). In contrast, the least active class included 65.3% of the population (class 1) and ranged from a weighted mean of 8.9 (Sunday) to 10.8 (Monday) minutes/day in MVPA across the week.

The weighted mean percent of MVPA bouts (Troiano) out of total wearing time per day ranged from 0% (class 1) to 6.7% (class 5; 0 to 53.3 minutes/day; Table 1). The least active class comprised 57.5% of the population and comprised all zeros for the weighted mean percent across the week (class 1; Figure 3). A variation of the weekend warrior class emerged for 8.2% of the population (class 2), with a low weighted mean percent of time spent in MVPA bouts for Monday through Thursday (0.0 to 0.2 minutes/day) compared to Friday (5.8 minutes/day), Saturday (10.2 minutes/day), and Sunday (13.3 minutes/day). The most active class represented 2.5% of the population and had much higher percent of time spent in MVPA bouts for every day of the week, ranging from 45.0 (Sunday) to 66.5 (Monday) minutes/day.

Using the lower Matthews cutpoint, the weighted mean percent of MVPA out of total wearing time per day ranged from 4.4% (class 1) to 39.5% (class 6; 34.6 to 266.3 minutes/day; Table 1). A small percent of the population (1.2%) were assigned to the most active class (class 6) and had a higher percent of MVPA out of total wearing time on the weekdays (328.7 (Friday) to 358.6 (Monday) minutes/day) compared to the weekends (226.9 (Saturday) to 237.0 (Sunday) minutes/day; Figure 4). A similar parallel class emerged with lower relative percents (class 5), ranging from 218.0 (Friday) to 255.1 (Tuesday) minutes/day on the weekdays compared to 129.0 (Sunday) to 152.7 (Saturday) minutes/day on the weekends. Overall, 3.8% of the population was assigned to a class with lower percents on the weekdays (178.6 (Wednesday) to 198.1 (Monday) minutes/day) but higher on the weekends (246.4 (Sunday) to 251.6 (Saturday) minutes/day; class 4). The least active class (class 1) included 24.3% of the population and the weighted mean percent of MVPA out of total wearing time ranged from 33.2 (Sunday) to 39.6 (Friday) minutes/day across the week.

The weighted mean percent of MVPA bouts (Matthews) out of total wearing time per day ranged from 0% (class 1) to 21.3% (class 5; 0.2 to 175.4 minutes/day; Table 1). The most active class comprised 5.6% of the population, with higher percents both on the weekdays (185.4 (Friday) to 212.4 (Tuesday) minutes/day) and weekends (109.8 (Sunday) to 124.1 (Saturday) minutes/day) compared to the other classes (Figure 5). The least active class (class 1) included 12.7% of the population with almost no time spent in MVPA bouts across all days of the week (0.0 to 0.3 minutes/day). A second class also with very few minutes in MVPA bouts emerged for another 17.0% of the population (3.7 (Thursday) to 5.1 (Monday) minutes/day on weekdays; 11.4 (Saturday) to 12.5 (Sunday) minutes/day on weekends; class 2).

Females comprised a higher percent of the least active class for weighted mean percent of MVPA and MVPA bouts (Troiano or Matthews) out of total wearing time (Table 2). Adults > =65 years comprised a higher percent of the least active class for percent of MVPA and MVPA bouts (Matthews) out of total wearing time. When comparing across classes, Hispanics comprised the highest relative proportion for the most active MVPA and MVPA bout classes (Troiano or Matthews).

### Sedentary behavior

The weighted mean percent of sedentary behavior out of total wearing time per day ranged from 35.0% (class 5) to 82.3% (class 1; 256.3 to 660.2 minutes/day; Table 1). All classes were stable across each day of the week except for the least sedentary group, which showed higher values on the weekends (336.7 (Sunday) to 346.5 (Saturday) minutes/day) compared to weekdays (255.2 (Thursday) to 292.4 (Friday) minutes/day; Figure 6). Overall, 31.4% of population was in the two most sedentary classes (class 1 mean 660.2 minutes/day; class 2 mean 546.8 minutes/day).

The weighted mean percent of sedentary bouts out of total wearing time per day ranged from 19.5% (class 7) to 85.6% (class 1; 159.0 to 746.7 minutes/day; Table 1). The class with the lowest percent of time spent in sedentary bouts comprised 20.3% of the population, with lower percents relative to the other classes across all days of the week (144.8 (Wednesday) to 166.0 minutes/day (Monday) on weekdays and 181.7 (Saturday) to 199.8 (Sunday) minutes/day on weekends; Figure 7). In contrast, 5.6% of the population was in the most sedentary class (749.0 (Wednesday) to 767.6 (Sunday) minutes/day across the week; class 1). A class emerged for 4.1% of adults wherein time spent in sedentary bouts was lower on the weekdays (186.6 (Thursday) to 288.2 (Friday) minutes/day) but higher on the weekends (441.9 (Sunday) to 551.3 (Saturday) minutes/day; class 6). An opposite class emerged for 8.7% of adults wherein time spent in sedentary bout was higher on the weekdays (420.9 (Friday) to 534.8 (Wednesday) minutes/day) but lower on the weekends (246.1 (Saturday) to 302.1 (Sunday) minutes/day; class 3).

Males represented a higher weighted mean percent of the class with the lowest percent of sedentary behavior out of total wearing time, but not for sedentary bouts (Table 2). The most sedentary and sedentary bout classes (class 1) were over represented by adults > =65 years. When comparing across classes, Hispanics comprised the highest relative proportion for the least sedentary and sedentary bout classes.

## Discussion

The LCA models provided a data reduction process to help identify patterns using minute-by-minute accelerometry data in order to explore meaningful contrasts. The models supported at least 5 or more distinct patterns for indicators of the total volume of physical activity (i.e., counts/minute), as well as MVPA and sedentary behavior both overall and in bouts. For both definitions of MVPA, the two least active classes represented the largest proportion of the population, and generally included a higher proportion of females and those 65 years and older. For sedentary behavior, most adults were assigned to the middle three sedentary classes (class 2 to 4).

As reported previously [8], participation in vigorous activity was low in the NHANES sample from 2003-2006, such that the LCA did not produce stable results when explored. This was also encountered by Metzger et al. [4] using 2003-2004 NHANES data on adults. Thus, we could only explore vigorous activity combined with moderate activity. In our analyses, we also explored other formulations of MVPA and MVPA bouts, including minutes/day and minutes/day controlling for total wearing time per day. We found that although wearing time did not affect the classification of MVPA very much, the MVPA time as a percent of wearing time was the best representation of this variable, since it accounted for total wearing time per day and most efficiently separated unique latent classes.

MVPA bouts (Troiano and Matthews) was particularly challenging to model correctly due to the skewness of the data, with a high proportion of adults not engaging in any MVPA bouts. To handle zero inflation and over dispersion, a LCA with zero-inflated negative binomial model was used. Future studies applying LCA to accelerometry should carefully assess the skewness of the data and when normality is violated, consider other types of modeling approaches.

Based on self-reported national data from 1999-2004, approximately 1% to 3% of adults belonged to the weekend warrior group [3]. This distinct pattern was subsequently confirmed using accelerometry from 2003-2004 NHANES data [4]. Using four years of NHANES data representing the US population, we also confirmed the weekend warrior pattern, identified among 3.2% of the sample (MVPA using the Troiano definition). Interestingly, the pattern of lower weekday and higher weekend for the total volume of physical activity was also identified for 9.1% of adults when viewing total counts/minute (class 5 and 6). Previously, Lee et al [2] found that men classified as weekend warriors from self-reported data had a lower risk of all-cause mortality when compared to sedentary men, particularly among those without major risk factors. Metzger et al. [5] found among adults that membership to the weekend warrior class was associated with a lower odds of the metabolic syndrome when compared to the least active class. The classes we derived can be used to explore these associations using NHANES data.

Although Hispanics have often self-reported low levels of MVPA relative to Non-Hispanic Whites when asked about leisure-time physical activity [13] or walking [14], our analyses indicated that Hispanics comprised a relatively larger proportion of the more active classes. Thus, Hispanics may accumulate more of their MVPA in activities other than during leisure, such as through active transportation and work activities.

Sedentary behavior, such as sitting, constitutes time spent in periods of little or no movement while awake, and at an energy expenditure ranging from 1.0-1.5 metabolic equivalents [15]. To our knowledge, this is the first paper to explore sedentary patterns among adults using LCA techniques. Of concern, the two most sedentary classes represented 31.4% of the population, with a weighted mean of 9.3 (class 2) to 12.4 (class 1) hours/day of sedentary behavior over the week. The least sedentary class that emerged had a relatively low percent of time spent in sedentary behavior on the weekdays but higher on the weekends (class 5). Even so, their percent of sedentary behavior was still lower on Saturdays and Sundays than the other four classes. When exploring bout minutes of sedentary behavior, several classes generally showed stable amounts throughout the week, though at different absolute percents. However, patterns also emerged with a lower percent of sedentary bouts out of total wearing time per day on the weekdays and more on the weekends (weekend couch potato), as well as higher percent of sedentary bouts out of total wearing time per day on the weekdays and fewer on the weekends (indicative of a weekend warrior pattern for sedentary behavior).

In our analyses, we also explored other formulations of sedentary behavior and sedentary bouts, including minutes/day and minutes/day controlling for sedentary wearing time. We found that wearing time greatly affected the classification of sedentary behavior and that representing the time as a percent of wearing time was the best representation of this variable to both account for wearing time and to maintain consistency throughout our analysis. Future use of this variable as an independent variable should also consider including accelerometer wear time as a potential confounder when appropriate.

### Limitations

These analyses are subject to several limitations. First, the uniaxial accelerometer used by NHANES under counts some activities, such as bicycling and weight lifting, and misses other activities, such as swimming, because the monitor was not waterproof and participants were told to remove it for any water-based activity. Second, the LCA models with sampling weights applied to these data assume data are missing at random. This assumption may not always be true, particularly when the accelerometer is removed for water activities. However, national data indicate that the proportion of adults who report swimming regularly is relatively low [16].

Third, the bootstrap likelihood ratio test we applied was based on unweighted data, such that it does not account for the sampling design in the test. However, we also used other criteria to make the final determination for the number of classes to use, including class sample size, substantive knowledge, and visual inspection. Fourth, it is possible that our latent class assignments still missed underlying patterns [4]. For example, there may be some workers whose weekend does not fall on Saturday or Sunday. The ordering of days could be explored differently, such as from least to most physical active, rather than from Monday to Sunday. Fifth, a strength is that our analyses resulted in latent class assignments that are available and can be used by others to address research questions (Additional file 3). The limitation is that this approach of deriving assignments separately from the modeling has lower statistical efficiency. However, we felt this trade-off was justified because assignments will remain stable to enhance comparability across future analyses.

## Conclusion

Using accelerometry data, this study identified patterns of overall physical activity, MVPA, and sedentary behavior from a national sample of adults. These findings can assist with intervention development to better understand how accelerometry-assessed physical activity and sedentary behavior are frequently patterned overall and by sociodemographic characteristics. Future NHANES analyses with these data can assess correlates of these patterns and associations with health outcomes. Moreover, exploration into whether the latent classes contribute over and above the absolute number of minutes for the same variable (counts/minute, MVPA, sedentary behavior) would help determine the further contribution of the patterning of the behavior.

There are also other possible uses of the LCA methods that could be applied to these data. For example, the methods can be used to develop clusters of health behaviors, including lack of physical activity as others have done using self-reported data [17]. These methods have also been applied to explore longitudinal patterns of self-reported leisure-time physical activity [18,19], walking [19], and bicycling [19] using an extension of LCA called latent class growth analysis. Another unique application combined self-report and accelerometry data to derive latent classes among a sample of youth [20]. These examples, along with our findings, offer exciting possibilities into studying physical activity patterns using detailed physical activity data.

## Additional files

Additional file 1:
**Weighted mean percents by day of week for latent classes derived from accelerometry among adults (n=7931); NHANES 2003-2006.**
Additional file 2:
**Wear time by the latent classes derived from accelerometry among adults (n=7931); NHANES 2003-2006.**
Additional file 3:
**Data dictionary for latent classes variables based on accelerometry measures among adults (NHANES 2003-2006).**

## Abbreviations

LCA: Latent class analysis
MVPA: Moderate to vigorous physical activity
NHANES: National Health and Nutrition Examination Survey
