# Supplementary material for: Physical activity and sedentary behavior patterns using accelerometry from a national sample of United States adults
Source: Int J Behav Nutr Phys Act. 2015 Feb 15;12:20. doi: 10.1186/s12966-015-0183-7 (PMC4336769; doi:10.1186/s12966-015-0183-7)
Supplement: Additional file 1: — Weighted mean percents by day of week for latent classes derived from accelerometry among adults (n=7931); NHANES 2003-2006. [file 12966_2015_183_MOESM1_ESM.pdf]

Additional File 1: Weighted mean percents by day of week for latent classes derived from accelerometry among adults (n=7931); NHANES 2003-2006

		Weighted Mean Percents by Day of Week						
	n	Monday	Tuesday	Wednesday	Thursday	Friday	Saturday	Sunday
Percent of MVPA (Troiano) out of total wearing time per day								
Class 1 - Least active	5410	1.3	1.2	1.1	1.2	1.2	1.2	1.1
Class 2	1768	4.4	4.4	4.5	4.3	4.2	3.7	3.3
Class 3	207	5.9	5.2	5.0	5.9	5.5	10.0	12.5
Class 4	473	9.1	9.3	9.7	9.4	8.1	5.8	4.8
Class 5 - Most active	73	17.5	16.1	19.2	16.3	16.0	14.5	12.4
Percent of MVPA bouts (Troiano) out of total wearing time per day								
Class 1 - Least active	4765	0.0	0.0	0.0	0.0	0.0	0.0	0.0
Class 2	569	0.0	0.0	0.0	0.0	0.7	1.2	1.7
Class 3	814	0.7	0.7	1.0	0.6	0.3	0.2	0.2
Class 4	1573	2.3	2.3	2.1	2.2	1.9	1.9	1.9
Class 5 - Most active	210	7.9	6.2	7.6	6.5	5.9	5.9	6.0
Percent of MVPA (Matthews) out of total wearing time per day								
Class 1 - Least active	2330	4.4	4.3	4.2	4.4	4.6	4.6	4.0
Class 2	3277	11.2	10.9	10.8	10.7	11.0	11.5	10.8
Class 3	1500	18.3	18.1	17.8	18.4	18.4	17.3	15.8
Class 4	245	23.3	21.1	20.7	21.2	23.1	31.4	31.7
Class 5	470	27.2	30.2	29.2	29.4	25.9	18.2	16.8
Class 6 - Most active	109	43.1	40.0	41.7	44.8	41.1	29.5	30.5
Percent of MVPA bouts (Matthews) out of total wearing time per day								
Class 1 - Least active	1233	0.0	0.0	0.0	0.0	0.0	0.0	0.0
Class 2	1410	0.6	0.4	0.5	0.4	0.5	1.4	1.5
Class 3	2827	3.0	2.8	2.5	2.7	2.7	3.5	3.3
Class 4	1997	8.7	8.0	8.4	8.1	8.2	8.6	7.8
Class 5 - Most active	464	22.4	24.8	22.4	24.8	22.4	15.0	13.8

Percent of sedentary behavior out of total wearing time per day								
Class 1 - Most sedentary	662	82.2	82.2	82.7	81.6	82.5	81.5	83.0
Class 2	2090	69.1	70.3	70.1	70.4	69.4	66.7	68.7
Class 3	2848	58.0	58.3	58.7	58.6	58.3	57.7	59.6
Class 4	1749	46.9	45.7	46.3	45.4	45.5	48.2	51.0
Class 5 - Least sedentary	582	32.6	31.5	31.5	30.8	34.5	43.5	45.2
Percent of sedentary bouts out of total wearing time per day								
Class 1 - Most sedentary	587	85.1	85.8	85.5	84.4	85.4	85.5	86.7
Class 2	1469	66.2	67.5	67.5	67.2	66.6	62.0	65.5
Class 3	656	50.3	58.1	60.9	59.7	46.4	29.5	37.1
Class 4	1449	49.7	47.9	46.7	47.3	50.3	57.9	58.7
Class 5	1951	35.5	34.1	36.4	35.7	35.5	32.7	39.7
Class 6	288	23.1	24.0	22.4	21.4	32.3	69.1	56.0
Class 7 - Least sedentary	1531	19.8	18.3	17.2	17.5	18.4	22.5	26.1

MVPA=moderate to vigorous physical activity
