# Supplementary material for: Physical activity and sedentary behavior patterns using accelerometry from a national sample of United States adults
Source: Int J Behav Nutr Phys Act. 2015 Feb 15;12:20. doi: 10.1186/s12966-015-0183-7 (PMC4336769; doi:10.1186/s12966-015-0183-7)
Supplement: Additional file 2: — Wear time by the latent classes derived from accelerometry among adults (n=7931); NHANES 2003-2006. [file 12966_2015_183_MOESM2_ESM.pdf]

Additional File 2: Wear time by the latent classes derived from accelerometry among adults (n=7931); NHANES 2003-2006

			Total Wear Time (hours/day)						
		Overall	Monday	Tuesday	Wednesday	Thursday	Friday	Saturday	Sunday
	n	Weighted Mean	Weighted Mean	Weighted Mean	Weighted Mean	Weighted Mean	Weighted Mean	Weighted Mean	Weighted Mean
Average counts/minute									
Class 1 - Least active	2088	14.2	14.4	14.6	14.4	14.4	14.5	14.2	14.0
Class 2	3372	14.1	14.5	14.6	14.5	14.4	14.5	13.8	13.5
Class 3	1596	14.0	14.3	14.2	14.4	14.3	14.4	13.8	13.3
Class 4	181	14.2	14.6	14.6	14.8	14.8	14.6	13.5	13.1
Class 5	571	13.7	14.1	14.1	14.0	13.9	14.1	13.6	12.8
Class 6 - Most active	123	13.6	14.0	14.4	13.7	13.6	13.7	13.2	13.2
Percent of MVPA (Troiano) out of total wearing time per day									
Class 1 - Least active	5410	14.1	14.4	14.4	14.4	14.3	14.4	13.9	13.6
Class 2	1768	14.2	14.5	14.6	14.6	14.5	14.6	13.8	13.4
Class 3	207	14.0	14.4	14.4	14.4	14.8	14.6	13.4	12.9
Class 4	473	13.8	14.2	14.1	14.2	14.0	14.2	13.7	13.2
Class 5 - Most active	73	13.6	14.4	14.5	13.9	13.7	13.9	12.8	12.4
Percent of MVPA bouts (Troiano) out of total wearing time per day									
Class 1 - Least active	4765	13.9	14.3	14.3	14.2	14.2	14.3	13.7	13.5
Class 2	569	14.4	14.4	14.7	14.7	14.6	15.0	14.2	13.5
Class 3	814	14.6	14.9	15.0	14.9	14.8	15.0	14.2	13.6
Class 4	1573	14.2	14.5	14.5	14.6	14.5	14.5	14.0	13.6
Class 5 - Most active	210	13.6	14.0	13.9	13.9	13.8	14.0	13.4	12.8
Percent of MVPA (Matthews) out of total wearing time per day									
Class 1 - Least active	2330	14.1	14.3	14.5	14.4	14.3	14.4	14.1	13.9
Class 2	3277	14.2	14.5	14.6	14.6	14.5	14.6	13.8	13.5
Class 3	1500	14.0	14.3	14.2	14.3	14.2	14.3	13.7	13.4
Class 4	245	14.0	14.4	14.6	14.5	14.6	14.3	13.4	13.1
Class 5	470	13.7	14.1	14.1	13.9	14.0	14.2	13.8	12.7
Class 6 - Most active	109	13.3	14.0	14.2	13.8	13.2	13.6	12.8	12.9

Percent of MVPA bouts (Matthews) out of total wearing time per day									
Class 1 - Least active	1233	13.7	14.0	14.0	13.9	13.9	14.0	13.8	13.5
Class 2	1410	14.2	14.5	14.7	14.6	14.4	14.6	13.9	13.6
Class 3	2827	14.2	14.5	14.6	14.6	14.5	14.6	13.9	13.6
Class 4	1997	14.0	14.4	14.4	14.5	14.3	14.4	13.8	13.4
Class 5 - Most active	464	13.7	14.0	14.3	13.9	13.8	14.0	13.6	13.0
Percent of sedentary behavior out of total wearing time per day									
Class 1 - Most sedentary	662	14.5	14.9	14.9	14.6	14.8	14.7	14.8	14.7
Class 2	2090	14.3	14.6	14.8	14.6	14.6	14.7	14.0	13.8
Class 3	2848	14.1	14.4	14.5	14.5	14.3	14.5	13.8	13.5
Class 4	1749	13.8	14.2	14.1	14.2	14.0	14.1	13.6	13.2
Class 5 - Least sedentary	582	13.4	13.8	13.8	13.7	13.7	13.9	13.3	12.5
Percent of sedentary bouts out of total wearing time per day									
Class 1 - Most sedentary	587	14.5	14.9	14.8	14.6	14.8	14.6	14.8	14.7
Class 2	1469	14.3	14.5	14.7	14.6	14.6	14.7	14.1	13.8
Class 3	656	14.3	14.7	15.0	14.7	14.7	14.9	13.6	13.4
Class 4	1449	14.4	14.7	14.8	14.7	14.6	14.7	14.1	13.8
Class 5	1951	14.0	14.3	14.3	14.4	14.2	14.4	13.8	13.4
Class 6	288	14.0	14.6	14.2	14.4	14.1	14.6	13.5	13.3
Class 7 - Least sedentary	1531	13.5	13.8	13.8	13.8	13.8	13.8	13.3	12.7

MVPA=moderate to vigorous physical activity
