# Supplementary material for: Physical activity and sedentary behavior patterns using accelerometry from a national sample of United States adults
Source: Int J Behav Nutr Phys Act. 2015 Feb 15;12:20. doi: 10.1186/s12966-015-0183-7 (PMC4336769; doi:10.1186/s12966-015-0183-7)
Supplement: Additional file 3: — Data dictionary for latent classes variables based on accelerometry measures among adults (NHANES 2003-2006). [file 12966_2015_183_MOESM3_ESM.pdf]

Additional File 3: Data dictionary for latent classes variables based on accelerometry measures among adults (NHANES 2003-2006)

Variable Name	SAS Label	SAS value
SEQN	Respondent sequence number	
ADHERENT	Indicator of participant adherent status	Categorical:<br>0 = Did not wear accelerometer<br>1 = Wore accelerometer but not calibrated or faulty<br>2 = Wore accelerometer but not adherent<br>3 = Adherent<br>. = under 18 years
**Latent classes for CPM**		
LC6_CNTMIN2	Latent classes on CPM	Categorical:<br>1 = Class 1<br>2 = Class 2<br>3 = Class 3<br>4 = Class 4<br>5 = Class 5<br>6 = Class 6
CNTMIN_PP1	Posterior probability in latent class 1 -- on CPM	Continuous
CNTMIN_PP2	Posterior probability in latent class 2 -- on CPM	Continuous
CNTMIN_PP3	Posterior probability in latent class 3 -- on CPM	Continuous
CNTMIN_PP4	Posterior probability in latent class 4 -- on CPM	Continuous
CNTMIN_PP5	Posterior probability in latent class 5 -- on CPM	Continuous
CNTMIN_PP6	Posterior probability in latent class 6 -- on CPM	Continuous
**Latent classes for % MVPA (Troiano)**		
LC_PCT_MVTO2	Latent classes on % MVPA (Troiano) out of total	Categorical:<br>1 = Class 1<br>2 = Class 2<br>3 = Class 3<br>4 = Class 4<br>5 = Class 5
PMVTO_PP1	Posterior probability in latent class 1 -- % MVPA (Troiano)	Continuous
PMVTO_PP2	Posterior probability in latent class 2 -- % MVPA (Troiano)	Continuous

PMVTO_PP3	Posterior probability in latent class 3 -- % MVPA (Troiano)	Continuous
PMVTO_PP4	Posterior probability in latent class 4 -- % MVPA (Troiano)	Continuous
PMVTO_PP5	Posterior probability in latent class 5 -- % MVPA (Troiano)	Continuous
**Latent classes for % MVPA bouts (Troiano)**		
LC_PCT_MVBTO2	Latent classes on % MVPA bouts (Troiano) out of total	Categorical:<br>1 = Class 1<br>2 = Class 2<br>3 = Class 3<br>4 = Class 4<br>5 = Class 5
PMVBTO_PP1	Posterior probability in latent class 1 -- % MVPA bouts (Troiano)	Continuous
PMVBTO_PP2	Posterior probability in latent class 2 -- % MVPA bouts (Troiano)	Continuous
PMVBTO_PP3	Posterior probability in latent class 3 -- % MVPA bouts (Troiano)	Continuous
PMVBTO_PP4	Posterior probability in latent class 4 -- % MVPA bouts (Troiano)	Continuous
PMVBTO_PP5	Posterior probability in latent class 5 -- % MVPA bouts (Troiano)	Continuous
**Latent classes for % MVPA (Matthews)**		
LC6_PCT_MVCM2	Latent classes on % MVPA (Matthews) out of total	Categorical:<br>1 = Class 1<br>2 = Class 2<br>3 = Class 3<br>4 = Class 4<br>5 = Class 5<br>6 = Class 6
PMVCM_PP1	Posterior probability in latent class 1 -- % MVPA (Matthews)	Continuous
PMVCM_PP2	Posterior probability in latent class 2 -- % MVPA (Matthews)	Continuous
PMVCM_PP3	Posterior probability in latent class 3 -- % MVPA (Matthews)	Continuous
PMVCM_PP4	Posterior probability in latent class 4 -- % MVPA (Matthews)	Continuous
PMVCM_PP5	Posterior probability in latent class 5 -- % MVPA (Matthews)	Continuous
PMVCM_PP6	Posterior probability in latent class 6 -- % MVPA (Matthews)	Continuous
**Latent classes for % MVPA bouts (Matthews)**		

LC6_PCT_MVBCM2	Latent classes on % MVPA bouts (Matthews) out of total	Categorical:<br>1 = Class 1<br>2 = Class 2<br>3 = Class 3<br>4 = Class 4<br>5 = Class 5
PMVBCM_PP1	Posterior probability in latent class 1 -- % MVPA bouts (Matthews)	Continuous
PMVBCM_PP2	Posterior probability in latent class 2 -- % MVPA bouts (Matthews)	Continuous
PMVBCM_PP3	Posterior probability in latent class 3 -- % MVPA bouts (Matthews)	Continuous
PMVBCM_PP4	Posterior probability in latent class 4 -- % MVPA bouts (Matthews)	Continuous
PMVBCM_PP5	Posterior probability in latent class 5 -- % MVPA bouts (Matthews)	Continuous
**Latent classes for % sedentary**		
LC_PCT_SD2	Latent classes on % sedentary out of total	Categorical:<br>1 = Class 1<br>2 = Class 2<br>3 = Class 3<br>4 = Class 4<br>5 = Class 5
PSD_PP1	Posterior probability in latent class 1 -- % Sedentary	Continuous
PSD_PP2	Posterior probability in latent class 2 -- % Sedentary	Continuous
PSD_PP3	Posterior probability in latent class 3 -- % Sedentary	Continuous
PSD_PP4	Posterior probability in latent class 4 -- % Sedentary	Continuous
PSD_PP5	Posterior probability in latent class 5 -- % Sedentary	Continuous
**Latent classes for % sedentary bouts**		
LC7_PCT_SDB2	Latent classes on % sedentary bouts out of total	Categorical:<br>1 = Class 1<br>2 = Class 2<br>3 = Class 3<br>4 = Class 4<br>5 = Class 5<br>6 = Class 6<br>7 = Class 7
PSDB_PP1	Posterior probability in latent class 1 -- % Sedentary bouts	Continuous
PSDB_PP2	Posterior probability in latent class 2 -- % Sedentary bouts	Continuous

PSDB_PP3	Posterior probability in latent class 3 -- % Sedentary bouts	Continuous
PSDB_PP4	Posterior probability in latent class 4 -- % Sedentary bouts	Continuous
PSDB_PP5	Posterior probability in latent class 5 -- % Sedentary bouts	Continuous
PSDB_PP6	Posterior probability in latent class 6 -- % Sedentary bouts	Continuous
PSDB_PP7	Posterior probability in latent class 7 -- % Sedentary bouts	Continuous

CPM=counts per minute; MVPA=moderate to vigorous physical activity
